# SPATIALLY RESOLVED MAPPING OF CELL-SPECIFIC SENESCENT PROFILES IN AGING MOUSE BRAIN

**DOI:** 10.1093/geroni/igac059.2212

**Published:** 2022-12-20

**Authors:** Chase Carver, Marissa Schafer

**Affiliations:** Mayo Clinic, Rochester, Minnesota, United States; Mayo Clinic, Rochester, Minnesota, United States

## Abstract

Cellular senescence is a conserved mechanism of aging characterized by cell cycle arrest and secretion of a senescence-associated secretory phenotype comprised of proinflammatory cytokines, chemokines, proteases, and growth factors that disrupt cellular homeostasis by promoting sterile inflammation and aberrant tissue remodeling. Tissue microenvironment, induction mechanism, and duration since senescence onset contribute to senescent cell heterogeneity, making identification challenging. Senescent cell contribution to age-related cognitive decline suggests that the hippocampus may be a key brain region for uncovering functional identities of senescence. Therefore, our goal is to contextually and spatially map senescent cells in comparison of cell-type specific morphological, transcriptional, and functional features that distinguish them among highly-ordered brain microenvironments. GeoMx Digital Spatial profiling was used to investigate cell identity and senescence markers in the aging brain to develop transcriptomic profiles from 22-24-month old mice. We also adapted Imaging Mass Cytometry with a curated panel of antibody-labeled proteins in fixed brain sections. We observed that senescent-like phenotypes of cells from myeloid and non-myeloid origin accumulate in the hippocampus and cortex, whereas CD45+ immune cells primarily accumulate in the white matter regions, in close proximity to microglia and GAL3+ cells. We profiled differences in ramified and ameboid microglia, in comparison of young and old mice, with emphasis on the chemotactic microenvironment involving activation of inflammatory signals. These advanced, multi-plex imaging modalities enable in-depth characterization of senescent cell features in the aging brain. Beyond morphological profiling, we demonstrate enhanced resolution and detection of individualized senescence in the aging brain.

